# Dentine sensitivity caused by illumination of intraoral scanner and light curing unit

**DOI:** 10.4317/jced.59251

**Published:** 2022-05-01

**Authors:** Prawnapa Natongkham, Pattaranat Banthitkhunanon, Sitthichai Wanachantararak

**Affiliations:** 1 Department of Prosthodontics, Faculty of Dentistry, Chiang Mai University, Chiang Mai, Thailand; 2 Department of Oral Biology and Oral Diagnosis Sciences, Faculty of Dentistry, Chiang Mai University, Chiang Mai, Thailand

## Abstract

**Background:**

Patients often compliant sensitivity to high-intensity light irradiated application during dental procedures. This study aims to investigate tooth sensitivity caused by high-intensity light irradiated from an intraoral scanner (IOS) and a light-curing unit (LCU).

**Material and Methods:**

Forty-five teeth from 45 healthy volunteers were included. These were equally classified into three groups using the cold test (4 ± 1 °C) and NRS pain assessment; A=control, B=cavity without sensitivity, and C=cavity with sensitivity. Two thermocouple probes were attached to the cervical area of the experimental and control tooth with a composite resin. Tooth sensitivity response by participant grip force was monitored. The digital oscilloscope was used to record two surface temperatures and a pain response during an IOS or a LCU irradiation. The high-intensity light from a LCU and an IOS was randomly applied at 2 mm above the cervical area for 20s. The data were compared statistical with two-way repeated measures ANOVA and Pearson’s correlation.

**Results:**

The illumination caused increasing surface temperatures of about 22.98 ± 3.20 °C for a LCU and 5.86 ± 1.46 °C for an IOS from a 29.5 °C baseline. As the light intensity from the LCU generated more heat, participants reported more tooth sensitivity with a shorter response time (2.10s to 18.70s). There was a positive correlation between surface temperature and pain response (R2 = 0.232; *p*<0.01).

**Conclusions:**

The heat from high-intensity light from a LCU and an IOS can cause tooth sensitivity in some individuals especially those who had a cervical cavity with dentine sensitivity. The higher light intensity would raise the surface temperature and cause a higher sensitivity response.

** Key words:**Light curing unit, intraoral scanner, tooth temperature, dentine sensitivity.

## Introduction

Dentine sensitivity was often located at the buccal cervical region in the premolars and canines, characterized by short and sharp pain that responded to non-noxious stimuli such as cold and hot drinks (1,2). After the loss of its cover, dentine becomes more sensitive to temperature changes which causes vigorous movement of dentinal fluid to activate mechanoreceptors in the deeper dentine and pulpal surface (3,4). The outward flow stimuli such as cold stimulus etc. can cause more tooth sensitivity response than an inward flow (5,6).

During dental treatment, patients occasionally complain of discomfort due to heat generated from instruments or materials used during treatment. High intensity light from LCU illumination to polymerize bonding agent and composite restoration can cause substantial tooth sensitivity in some cases (7). In addition, some reports identified tooth sensitivity to the use of modern introduced technology, such as an IOS which generates high-intensity light which also produces heat (8). Some research identifies the possibility that the heat caused by these high intensity illuminations not only stimulates fluid movement through dentine but could also raise pulpal temperature (9,10) that may then produce pain.

However, the dentine sensitivity responded to heat developing during high intensity light illumination from dental instruments has never been studied and any underlying mechanism still remains unclear. Therefore, the objective of this study was to investigate how high-intensity light-emitting caused dentine sensitivity in humans by monitoring the temperature change on tooth surfaces of normal, cervical cavity without sensitivity and cervical cavity with sensitivity teeth.

## Material and Methods

This study has been approved by the Human Experimentation Committee, Faculty of Dentistry, Chiang Mai University, Thailand, certificate number 38/2020. Forty-five patients (17 males and 28 females) aged between 23 and 59 years with good general health and oral hygiene were participants for this research. All participants were instructed prior to commencement to give their consent to attend and participate in this research experiment. Any participants prone to have tooth sensitivity such as the teeth under orthodontic treatment, desensitizing, tooth bleaching treatments, or abutment of the partial prosthesis and patients with anorexia or bulimia conditions were excluded.

Every subject was examined by a clinician using inspection for a cervical cavity at the buccal surface of canine and premolar and cold test to evaluate tooth sensitivity response. Two drops of 4 ºC water (about 0.0238 ml) from a 25 gauge needle attached to a 5ml syringe were applied onto the cervical area and asked for a response in a numeric rating scale of 0-10 (NRS). The participants were classified into three groups according to cervical cavity and tooth sensitivity response to the cold test. Group A, the normal cervical area with a tooth sensitivity score < 4. Group B, 1.0-1.5 mm depth cervical cavity with tooth sensitivity score < 4. Group C, 1.0-1.5 mm depth cervical cavity with tooth sensitivity score of 5 and above (11).

**Figure 1:**
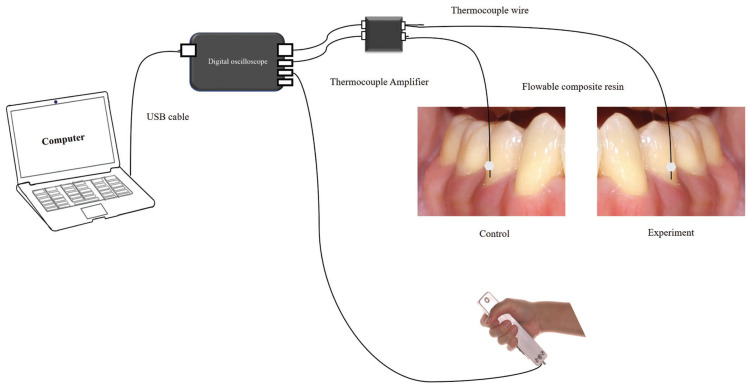
The diagram demonstrated the experimental setup. The two thermocouple probe tips were attached to the cervical area of the experimental and control tooth with composite resin. The digital oscilloscope was used to record two surface temperatures and pain responses during an IOS or a LCU irradiation.

The temperature change at the cervical tooth surface of experimental and control teeth was monitored using two 0.5 mm diameter tip of type K thermocouple probes (Omega, Stamford, United States) and the room temperature was controlled at 23 ± 1 °C throughout the experiment. The neck of the thermocouple probe was fixed with flowable composite resin (FiltekTM Supreme, Seefeld, Germany) to the tooth surface at level 1 mm above the gingival margin. The tip of the thermocouple probe was coated with nail varnish to provide electrical insulation from the environment. The alternative voltage of the thermocouple probe caused by temperature change was amplified by thermocouple amplifier SEN30101/K1-5V0 (Playing with Fusion, Portland, United States) to display and store data on a laptop computer using a digital oscilloscope (Hantek® 6000B, Qingdao, China) for further analysis (Fig. 1).

The participant’s tooth sensitivity perception was real - time monitoring using a hand-held squeezing device made from stainless-steel fork equipped with full bridge strain gauges. The squeezing force was amplified to display and store simultaneously on the same oscilloscope and laptop computer. Before the experiment, the participants were trained to be familiar with giving their responses using this device. Full grip strength squeezing represents highest imagination of tooth sensitivity or pain (score 10), while no squeezing means no pain (score 0). The squeezing force was recorded during the experiment and later converted into a tooth sensitivity score compared with full grip strength.

Two high-intensity light illumination machines; IOS (PRIMESCAN®, Sirona, Bensheim, Germany) and LCU (Bluephase N®, Ivoclar Vivadent, Lichtenstein, Germany) were randomly shined on the cervical area of the experimental tooth for 20s, and then having a 3 minutes break interval. The tips of these instruments were kept 2 mm apart from the tooth surface with thermocouple probe. After finishing the experiment, the cervical cavity was restored with composite resin (FiltekTM Z350 XT, Seefeld, Germany) as a complimentary procedure from this study.

The light intensity from a LCU (high - intensity mode) and an IOS was measured by using a BA Optima light meter (B.A. International, Northampton, United Kingdom). Evaluation of irradiance: ten measurements for each instrument were conducted at irradiation times of 10s, 20s, and 30s, respectively.

The pulpal temperature changes during the illumination of two instruments were investigated on three extracted human premolars, *in vitro*. The 1.5 mm depth cervical cavity was prepared at the buccal cervical area at level 1 mm above the cementoenamel junction (CEJ) using a round diamond bur attached to a high-speed airotor handpiece with water spray cooling. The root was cut off at the level of 2 mm apically to the CEJ for inserting a thermocouple probe in a plastic hose. The remaining pulpal tissue was removed through the cut end. The tip of the probe, coated with nail varnish, was placed to point to the cervical cavity and confirmed with a radiograph. Two additional thermocouple probes were attached to the buccal and lingual surface of the crown with composite resin. The surrounding temperature was controlled at 23 ± 1ºC. The light from a LCU and an IOS were illuminated on the buccal cavity, similar to the *in vivo* study. The temperature change was recorded similarly with the computer for further analysis.

The data were analyzed with two-way repeated measures ANOVA and Pearson correlation to investigate the effect of temperature change on the tooth surface and tooth sensitivity response from two instruments *in vivo*. The *P* value less than 0.05 was considered a significant difference.

## Results

The two devices used in this study produced substantial heat, raising up the surface temperature significantly. The illumination of LED from LCU raised significantly higher surface temperature than the IOS (*p* < 0.001). The LCU illumination could raise surface temperature to 49.94 ± 2.85 to 51.67 ± 3.39ºC while an IOS illumination increased surface temperature to 34.55 ± 1.60 to 35.31 ± 0.87 ºC from 29.5ºC of oral temperature baseline. The temperature change at tooth surface was shown in Table 1.

**Table 1:**
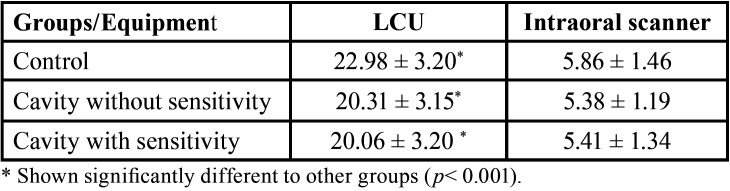
The mean ± SD of temperature change from oral temperature (29.5oC) during the 20s irradiation of a light curing unit and intraoral scanner.

The *in vitro* investigation of LCU irradiation showed that the surface temperature raised immediately after starting irradiation and continuous raising to 51.75 ± 2.01°C at 20s, but the pulpal temperature underneath gradually went up to 27.89 ± 0.94 °C which increased by less than 5°C. However, the pulpal temperature caused by the irradiation of an IOS was negligible as the surface temperature rose up about 5°C.

**Figure 2:**
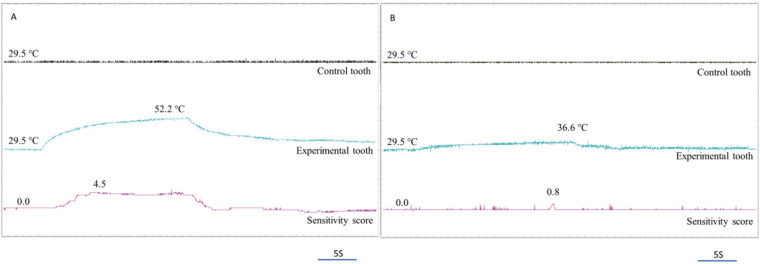
The samples of real-time recordings indicated temperature change at the cervical region of the experimental and control teeth, together with the tooth sensitivity response in the same participant. (A) Illumination with LCU resulted in a high increase in surface temperature to 52.2°C and tooth sensitivity score of 4.5 out of 10 for participants in the cervical cavity with sensitivity group. (B) Illumination with IOS was a slight increase in surface temperatures and tooth sensitivity responded.

Not only did higher tooth sensitivity score respond to LCU irradiation, but also a greater number of participant responses. The tooth sensitivity response from participants in a cavity with sensitivity group was 5.10 ± 1.96, significantly greater than participants in the cavity without sensitivity and control group, which were 4.43 ± 2.62 and 3.29 ± 0.27. All participants from the cervical cavity with sensitivity group (n = 15) reported tooth sensitivity to LCU irradiation, while some participants in control (n = 3) and cervical cavity without sensitivity (n = 6) groups indicated tooth sensitivity. The cervical cavity with sensitivity group had a response time to irradiate of LCU (2.10 - 18.70s) shorter than the cervical cavity without sensitivity (6.30 - 19.30s), and control (7.56 - 12.8s). In addition, seven participants responded to the irradiated IOS with fewer tooth sensitivity responses (1.78 ± 1.20) (Fig. 2).

The LCU, high - intensity mode, had an average light intensity stronger than an IOS. The average light intensities from LCU with 10s, 20s, and 30s irradiation periods were 1487.60 ± 13.90, 1467.40 ± 10.50, and 1454.00 ± 14.31 mW/cm2, was decreased respectively. Whereas, the light intensity emitted from an IOS tended to increase (327.0 ± 9.81, 333.50 ± 9.91, and 347.90 ± 9.99 mW/cm2).

**Figure 3:**
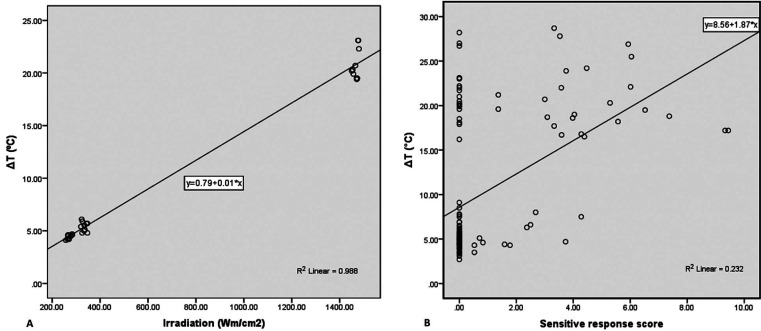
The scatter plot shows that the temperature increase was strongly correlated with the light intensity (R2 = 0.988) (A) and also with the tooth sensitivity score (B).

Pearson analysis suggested that the changing of surface temperature had strongly positive correlation with light emission intensity (R2 = 0.988, *p* < 0.01), and positive correlation with tooth sensitivity response (R2 = 0.232; *p* < 0.01) (Fig. 3).

## Discussion

The high-intensity mode of LCU irradiated 1467.40 mW/cm2 of light produced the highest heat on the tooth surface in this study, similar to Gomes *et al*. in 2013, and Mouhat *et al*. in 2017 (12,13). This would explain the patient discomfort or tooth sensitivity during the LCU to polymerization composite restoration, especially when using high-intensity mode (7). Even though polymerization of composite resin has an exothermic reaction (14,15), this study provided evidence to confirm that the high light intensity itself could produce heat enough to cause dentine sensitivity, even the high - intensity mode LCU used in this study might produce more heat than the average intensity light LCU at 1200 mW/cm2 (16).

The use of an IOS offers faster, more comforTable, and patient preference to conventional impression techniques (17,18). However, it raises surface temperature about 5.86°C which may be enough to cause tooth sensitivity in some cases, especially those who had cervical cavity with sensitive dentine. In addition, another IOS (TRIOS 3®, 3 shape, Copenhagen, Denmark) caused increase in surface temperature of about 4.88 ± 1.13°C, and some participants reported tooth sensitivity. The tooth sensitivity related to the use of IOS has not been reported elsewhere, nor tooth sensitivity response to the 2-3°C exothermic reaction during polymerization of conventional elastomeric materials (19).

The heat generated from high light intensity of an IOS and a LCU in this study was only the variable for investigation while the duration of illumination and distance was fixed that followed factors associated with heat development by Mouhat *et al*. (13). The tooth surface temperature had a stronger positive correlation with the light intensity from a LCU and an IOS (R2 = 0.988) than Gomes *et al*. study (12). The LCU, which had higher light intensity, raised high surface temperature more than an IOS. Moreover, Pearson analysis suggested that there was a positive correlation between surface temperature and tooth sensitivity response (R2 = 0.232) similar to Mengle *et al*. in 1993 that caused mild pain perception (20).

The hydrodynamic theory suggested that the thermal stimuli cause turbulence of fluid flow to activate the strength receptor located at the pulp-dentine complex. Similar to other hot stimuli, the heat generated from intense light sources activated the inward movement of dentinal fluid and stimulated pain mechanoreceptor in the pulp (21,22). The stimulus applied on thinner dentine which has higher hydraulic conductance would cause more vigorous dentinal fluid movement effect on the receptor (4,23).

An alternative explanation for the tooth sensitivity response to the elevation of tooth surface temperature was specific heat receptors or heat fibers involved in a different transduction mechanism (24). Heat - sensitive receptors located at the deeper dentine or surface of the pulp has been activated by heat that travels through dentine and raising the surrounding temperature. The TRPV3 and TRPV4 receptors responded to non-painful warmth stimuli (33-39°C), while TRPV1 and TRPV2 receptors could be activated at temperatures > 43°C and > 52 °C, respectively (25). As dentine is not a good thermal insulator, it had high porosity filled with dentinal fluid (26), the thinner dentine in the prepared tooth was prone to temperature change than thicker dentine (27). Our result from *in vitro* investigation showed that the illumination of LCU caused increasing in the pulpal temperature in the cervical cavity tooth at about 4.49 ± 0.94°C which coincided with previous studies (9,13). This elevation of temperature could possibly stimulate TRPV3 and TRPV4 receptors but could not explain the tooth sensitivity caused by the use of an IOS which only slightly raised pulpal temperature.

The cervical cavity with sensitivity group had more participants who reported tooth sensitivity to the light intensity of two light sources and a higher tooth sensitivity score than other groups. The results agreed that applying stimuli to the sensitive dentine caused more pain responses (3). According to hydrodynamic theory, the sensitive dentine has higher hydraulic conductance than non-sensitive dentine and enamel intact teeth (28,29). However, in case of a deep dentine cavity or less remaining dentine thickness, the use of these devices in any procedures requires technicians to be aware of the possibility of causing more pain. Further investigation is still needed.

## Conclusions

The heat generated by the illumination of LCU and IOS could cause tooth sensitivity in some individuals. In addition, the devices with higher light intensity would raise the surface temperature and cause more pain and discomfort. Caution should be taken, using these devices in the clinic on a non-anesthetized tooth with dentin hypersensitivity.
